# Traumatic spinal cord injury in South Korea for 13 years (2008–2020)

**DOI:** 10.1038/s41598-024-57965-4

**Published:** 2024-04-09

**Authors:** Sung Hyun Noh, Eunyoung Lee, Kyoung-Tae Kim, Sang Hyun Kim, Pyung Goo Cho

**Affiliations:** 1 Department of Neurosurgery, Ajou University Hospital, Ajou University School of Medicine, Suwon, 164 Republic of Korea; 2 Department of Neurosurgery, Yonsei University College of Medicine, Seoul, Korea; 3 Department of Neurology, McGovern Medical School at UTHealth, Houston, TX USA; 4 Department of Neurosurgery, School of Medicine, Kyungpook National University Hospital, Kyungpook National University, Daegu, Republic of Korea

**Keywords:** Neuroscience, Neurology, Signs and symptoms

## Abstract

Traumatic spinal cord injury (TSCI) has significant physical, psychological, and socioeconomic impacts. However, the epidemiological characteristics and treatment patterns of TSCI in South Korea remain unclear. This study aimed to investigate TSCI incidence and treatment behaviors in South Korea from 2008 to 2020. We included data from 30,979 newly diagnosed TSCI patients obtained from the Health Insurance Review and Assessment Service (HIRA). Treatment trends, location of surgery, surgical method, comorbidities, factors affecting hospital stay, and risk factors affecting readmission were analyzed. Patients were divided into the surgery group [n = 7719; (25%)] and the non-surgery group [n = 23,260; (75%)]. Surgical cases involved cervical (64%), thoracic (17%), and lumbar/sacral (19%) lesions. Anterior fusion (38%), posterior fusion (54%), and corpectomy (8%) were the surgical methods. Surgical treatments increased annually. Factors influencing hospital stay included male sex, older age, and higher Charlson comorbidity index (CCI). Female sex and higher CCI scores were associated with readmission. In conclusion, a quarter of all TSCI patients underwent surgery, with an upward trend. Risk factors for longer hospital stays were thoracic spine injury, older age, higher CCI, and male sex. Risk factors for readmission included age range of 40–59 years, lumbar/sacral spine injuries, CCI score of 2, and female sex.

## Introduction

Traumatic spinal cord injury (TSCI) is a complete or incomplete paraplegia/tetraplegia that can lead to severe lifelong dysfunction and can be life-threatening owing to recurrent complications such as pneumonia, severe pressure sores, and urinary tract infections. Despite improvements in the initial treatment and management strategies, TSCI remains a fatal event, leading to severe and permanent disability and death, placing a significant burden on society.

Some studies have compared the prevalence and incidence of acute SCI under various geographical and economic conditions. Acute SCI accounts for 2.6% of cases in major trauma centers in North America^1^. Not only are there more than 10,000 new patients each year, but this number is even higher if those who died before reaching the hospital are included^2^. Canada had an incidence of 3.6 per million^3^, whereas Ireland had 195.4 per million^4^. The estimated incidence of TSCI in Japan in 2018 was 49 cases per million people^5^.

Many TSCI epidemiological studies have been conducted in South Korea^6–8^. Han et al. reported that the number of patients with spinal cord injury was 5.59% (2,726,910 persons) in Korea at 2014^6^. Shin et al. compared and analyzed trends in traumatic spinal cord injury and non-traumatic spinal cord injury between 1987–1996 and 2004–2008^7^. The TSCI rate decreased in 2004–2008 compared to 1987–1996^7^. Most recently, Choi et al. investigated acute SCI in South Korea from 2007 to 2017 in 2019 and reported that incidence was 26.4 per million^9^. Although studies focusing on SCI are currently being conducted in South Korea, no previous studies have focused on TSCI surgery in the South Korean population. This study aimed to analyze the prevalence and treatment methods of TSCI in South Korea over the past 13 years. In addition, we analyzed the risk factors affecting readmission and hospitalization periods. To the best of our knowledge, this is the first study to evaluate TSCI surgical treatment method using HIRA data.

## Methods

### Study design and data source

This study was approved by the Institutional Review Board of Ajou university hospital (2021-01-009). All the participants provided written informed consent to participate in the study. All methods were performed in accordance with the relevant guidelines and regulations by including a statement. This retrospective population-based cohort study used data from the Health Insurance Review and Assessment Service (HIRA) database. South Korea's national health insurance system, which was established in 1989, is run by the government and covers medical expenses for outpatient, inpatient, and emergency medical services. All the hospitals and clinics in South Korea are required to provide the HIRA with information about the diagnosis, treatment, and clinical behavior of outpatients and inpatients to bill the HIRA. Therefore, in addition to personal information such as the patient’s age and sex, all information about diagnosis, tests performed, and treatment can be obtained through HIRA data^10^.

### Data collection and variables

This study enrolled 30,979 patients with acute TSCI from the HIRA database between January 1, 2008, and December 31, 2020. Inclusion criteria were patients who underwent surgery to treat traumatic spinal cord injury. Exclusion criteria were patients who had suffered a traumatic spinal cord injury in the past 3 years (2005–2007) and had undergone spinal surgery due to degenerative spinal disease, infection, tumor, or inflammatory disease. We divided the patients into two groups: 7719 patients in the surgery group and 23,260 patients in the non-surgery group.

Operation codes have been standardized for medical billing in the HIRA. The variables were identified using the International Classification of Diseases version 10 (ICD-10) codes. TSCI included injury of nerves and spinal cord at the neck level (code: S14), injury of nerves and spinal cord at the thoracic level (code: S24), injury of the lumbar and sacral spinal cord, and nerves at the abdomen, lower back, and pelvis level (code: S34). ICD codes of TSCI surgery included cervical corpectomy and fusion (code: N0451), thoracic corpectomy and fusion (code: N0452), lumbar corpectomy and fusion (code: N0453), anterior cervical fusion (codes: N2461, N0464, N1491), anterior thoracic fusion (codes: N2465, N0465), anterior lumbar fusion (codes: N1466, N0466), posterior cervical fusion (codes: N2467, N2468, N0467), posterior thoracic fusion (codes: N0468, N1492), posterior lumbar fusion (codes: N0469, N1460, N1469, N2470), posterior sacrum fusion (code: N0593), cervical laminectomy (codes: N2497, N1497), thoracic laminectomy (codes: N1498, N2498), and lumbar laminectomy (codes: N2499, N1499). The surgical code counting was performed with all surgical codes prescribed for a given patient.

Comorbidity was assessed using the Modified Charlson Comorbidity Index (CCI) presented by Quan et al.^5^. Comorbidity was defined as three or more outpatient clinic visits or hospitalization for at least 2 days at a given diagnosis according to the primary disease code for the year of enrollment^4^. Age was divided into five groups: < 20, 20–39, 40–59, 60–79, and ≥ 80 years old. The hospitals in which patients were first treated were classified as tertiary hospitals, general hospitals, hospitals, and clinics. The residents were grouped into 16 categories based on their registered addresses. Health insurance eligibility was classified into two categories: national health insurance and medical aid. The length of the hospital stay was also investigated. Readmission was defined as hospitalization with the same diagnosis within 90 days after surgery. Participants were classified into the surgery and the non-surgery groups for comparative analysis.

### Statistical analysis

Pearson’s Chi-squared test for categorical variables and Wilcoxon rank-sum test for continuous variables were used to investigate the differences between the treatment in the non-surgery and surgery groups. The trend in the number of surgeries performed by each surgical method, corpectomy fusion, anterior fusion, posterior fusion, and laminectomy, was tested by the Cochran-Armitage trend test. The percentage of TSCI patients who underwent surgery was also examined. Univariable and multivariable linear regression analyses were performed to investigate the factors associated with the length of stay at admission for TSCI. Moreover, univariable and multivariable logistic regression analyses were conducted to assess any risk factors associated with readmission after discharge. Covariates include sex, diagnosis, age group, hospital class and location, insurance type, and CCI scores. All covariates in the univariable analysis were included in the multivariable analysis to adjust for confounding factors. All statistical analyses were performed using SAS (version 9.4, SAS Institute, Cary, NC, USA). Statistical significance was set at P < 0.05.

## Results

### Baseline characteristics of TSCI patients from 2008 to 2020 in South Korea

A total of 30,979 patients [20,947 (67.6%) men and 10,032 (32.4%) women] with a mean age of 52.5 ± 10.3 years were evaluated. Table 1 describes the baseline characteristics of patients with TSCI. Most patients were aged 40–59 years, followed by those aged 60–79, 20–39, over 80, and under 19 years. TSCI was most commonly diagnosed in the neck (69.1%), followed by the abdomen, lower back and pelvis (22%), and the thorax level (8.9%). The most common surgical location was the cervical spine (64.4%), followed by the lumbar spine (18.3%), thoracic spine (17.2%), and sacrum (0.1%). The most common surgical method was anterior fusion (37.8%), followed by laminectomy (32.7%), posterior fusion (20.6%), and corpectomy fusion (8.9%).

**Table 1:** Baseline characteristics of spinal cord injury patients from 2008 to 2020 in South Korea.

Characteristics	All (n = 30,979)	Treatment without surgery (n = 23,260)	Treatment with surgery (n = 7719)	*P*
Sex, n (%)				< 0.0001
Male	20,947 (67.62)	15,117 (64.99)	5830 (75.53)	
Female	10,032 (32.38)	8143 (35.01)	1889 (24.47)	
Diagnosis—injury of nerves and spinal cord (ICD-10 code), n (%)				< 0.0001
At neck level (S14.x)	21,390 (69.05)	15,655 (67.30)	5735 (74.30)	
At thorax level (S24.x)	2772 (8.94)	1838 (7.90)	934 (12.10)	
At abdomen, lower back and pelvis level (S34.x)	6817 (22.01)	5767 (24.80)	1050 (13.60)	
Surgical method^a^, n (%)				0.4356
Corpectomy fusion	–	–	404 (5.23)	
Anterior fusion	–	–	3144 (40.73)	
Posterior fusion	–	–	1512 (19.58)	
Laminectomy	–	–	2659 (34.46)	
Location of surgery, n (%)				0.4598
Cervical	–	–	6140 (64.47)	
Thoracic	–	–	1633 (17.15)	
Lumbar	–	–	1742 (18.29)	
Sacrum	–	–	9 (0.09)	
Age group, n (%)				< 0.0001
–19	806 (2.60)	680 (2.92)	126 (1.63)	
20–39	4697 (15.16)	3789 (16.29)	908 (11.76)	
40–59	12,757 (41.18)	9694 (41.68)	3063 (39.68)	
60–79	10,956 (35.37)	7714 (33.16)	3242 (42.00)	
80–	1763 (5.69)	1383 (5.95)	380 (4.93)	
Hospital, n (%)				< 0.0001
Tertiary hospital	8938 (28.85)	4738 (20.37)	4200 (54.41)	
General hospital	11,240 (36.28)	8283 (35.61)	2957 (38.31)	
Hospital	6730 (21.72)	6187 (26.60)	543 (7.03)	
Clinic, nursing hospital	4071 (13.15)	4052 (17.42)	19 (0.25)	
Hospital region, n (%)				< 0.0001
Seoul	6282 (20.28)	4583 (19.7)	1699 (22.01)	
Busan	2125 (6.86)	1578 (6.78)	547 (7.09)	
Incheon	1595 (5.15)	1097 (4.72)	498 (6.45)	
Daegu	1758 (5.67)	1292 (5.55)	466 (6.04)	
Gwangju	1555 (5.02)	941 (4.05)	614 (7.95)	
Daejeon	1175 (3.79)	838 (3.60)	337 (4.37)	
Ulsan	586 (1.89)	440 (1.89)	146 (1.89)	
Gyeonggi	5392 (17.41)	4122 (17.72)	1270 (16.45)	
Gangwon	1259 (4.06)	936 (4.02)	323 (4.18)	
Choongbuk	934 (3.01)	727 (3.13)	207 (2.68)	
Choongnam	964 (3.11)	717 (3.08)	247 (3.20)	
Jeonbuk	2112 (6.82)	1555 (6.69)	557 (7.22)	
Jeonnam	1022 (3.3)	887 (3.81)	135 (1.75)	
Gyeongbuk	1772 (5.72)	1472 (6.33)	300 (3.89)	
Gyeongnam	2094 (6.76)	1795 (7.72)	299 (3.87)	
Jeju	340 (1.1)	266 (1.14)	74 (0.96)	
Sejong	14 (0.05)	14 (0.06)	0 (0.00)	
Insurance type, n (%)				0.0041
National health insurance	30,787 (99.38)	23,133 (99.45)	7654 (99.16)	
Medical aid	192 (0.62)	127 (0.55)	65 (0.84)	
Charlson Comorbidity Index, n (%)				0.7633
0	11,031 (35.61)	8267 (35.54)	2764 (35.81)	
1	6357 (20.52)	4751 (20.43)	1606 (20.81)	
2	4642 (14.98)	3505 (15.07)	1137 (14.73)	
$\ge$ 3	8949 (28.89)	6737 (28.96)	2212 (28.66)	
Length of stay, days, median [IQR]	22 [10, 77]	18 [8, 50]	50 [21, 193]	< .0001

*ICD-10* International Classification of Disease, 10th revision.

^a^Different type of surgical methods could be performed on one patients, so total number of surgical methods is 9524.

There were 23,260 patients (75%) in the non-surgery group and 7,719 patients (25%) in the surgery group. In the non-surgery group, the highest age group was 40–59 years, while in the surgery group, the highest age group was 60–79 years.

The non-surgery group received treatment at a general hospital, while the surgery group received therapy at a tertiary hospital. Hospitals where both groups were treated were mostly located in Seoul and Gyeonggi-do. In both groups, the most common CCI score was 0, followed by 3 or more. The median duration of hospitalization was 18 days in the non-surgery group and 50 days in the surgery group; the difference was statistically significant (P < 0.0001).

### Number of performed surgeries, their method, and yearly trend for each surgical method

Table 2 shows the trend in surgery according to the surgical method from 2008 to 2020. In 2008, there were 535 cases; in 2020, there were 915 cases. The number of surgeries gradually increased. The performed surgeries included corpectomy and fusion, anterior fusion, posterior fusion, and laminectomy, all of which showed a gradual increase and were statistically significant (P < 0.05).

**Table 2:** Number of performed surgery and its method and yearly trend for each surgical method.

Year	Corpectomy fusion		Anterior fusion		Posterior fusion		Laminectomy		All
	N	%	N	%	N	%	N	%	
2008	62	11.59	174	32.52	145	27.1	154	28.79	535
2009	68	12.66	187	34.82	137	25.51	145	27	537
2010	74	11.64	227	35.69	156	24.53	179	28.14	636
2011	63	10.66	219	37.06	142	24.03	167	28.26	591
2012	66	11.19	245	41.53	117	19.83	162	27.46	590
2013	64	9.52	266	39.58	135	20.09	207	30.8	672
2014	61	8.13	325	43.33	145	19.33	219	29.2	750
2015	68	8.92	301	39.5	151	19.82	242	31.76	762
2016	57	7.01	328	40.34	142	17.47	286	35.18	813
2017	64	7.1	372	41.24	140	15.52	326	36.14	902
2018	79	8.73	341	37.68	169	18.67	316	34.92	905
2019	65	7.1	294	32.1	190	20.74	367	40.07	916
2020	64	6.99	316	34.54	194	21.2	341	37.27	915
Total	855		3595		1963		3111		9524

Data were presented as N (%).

### Number of patients who underwent surgery and yearly trend

From 2008 to 2020, the trends in the surgery and non-surgery groups were analyzed (Table 3). In 2008, the proportions of patients in the surgery and non-surgery groups were 21.5% and 78.5%, respectively, whereas in 2020, it was 31% and 69%, respectively.

**Table 3:** Number of patients who underwent surgery and yearly trend.

Year	N	Treatment without surgery	Treatment with surgery
2008	1984	1557 (78.48)	427 (21.52)
2009	2098	1655 (78.88)	443 (21.12)
2010	2226	1733 (77.85)	493 (22.15)
2011	2109	1640 (77.76)	469 (22.24)
2012	2338	1861 (79.60)	477 (20.40)
2013	2388	1853 (77.60)	535 (22.40)
2014	2540	1939 (76.34)	601 (23.66)
2015	2528	1911 (75.59)	617 (24.41)
2016	2712	2037 (75.11)	675 (24.89)
2017	2609	1858 (71.22)	751 (28.78)
2018	2574	1827 (70.98)	747 (29.02)
2019	2498	1751 (70.10)	747 (29.90)
2020	2375	1638 (68.97)	737 (31.03)
All	30,979	23,260 (100)	7719 (100)

### Length of stay at admission for traumatic spinal cord injury and its associated factor from regression analysis

Factors affecting hospital stay after TSCI are presented in Table 4. The hospitalization period in the surgery group was 102 days longer than that in the non-surgery group, and the hospitalization period was 19 days longer in men than in women. Compared to lumbar and sacral injuries, patients with cervical spine injuries had a longer hospital stay of 59 days, and those with thoracic injuries had a longer hospital stay of 127 days. In terms of age, the hospitalization period was longer as age increased, and the hospitalization period was the longest in tertiary general hospitals. Hospital stays were 253 days longer in those using medical aid than in those with health insurance. As regards CCI, patients with a score of 3 or higher had the longest hospital stay.

**Table 4:** Length of stay at admission for traumatic spinal cord injury and its associated factor from regression analysis.

Parameter	Univariate regression analysis				Multivariate regression analysis			
	Estimate	Std	t-value	*P*	Estimate	Std	t-value	*P*
Treatment with surgery	102.82	4.55	22.60	< 0.0001	94.90	4.92	19.29	< 0.0001
Treatment without surgery								
Male	18.70	4.24	4.41	< 0.0001	16.57	4.36	3.80	< 0.0001
Female								
Dx: S14.x	58.81	4.83	12.17	< 0.0001	49.84	5.20	9.59	< 0.0001
Dx: S24.x	126.64	7.83	16.18	< 0.0001	91.02	7.94	11.46	< 0.0001
Dx: S34.x								
Age: –19								
Age: 20–39	31.57	13.27	2.38	0.0174	26.06	13.07	1.99	0.0463
Age: 40–59	53.73	12.64	4.25	< 0.0001	33.78	12.51	2.70	0.0069
Age: 60–79	91.16	12.70	7.18	< 0.0001	51.48	12.74	4.04	< 0.0001
Age: 80–	111.40	14.80	7.53	< 0.0001	72.13	14.84	4.86	< 0.0001
Tertiary hospital	34.71	6.59	5.27	< 0.0001	−36.01	7.19	−5.01	< 0.0001
General hospital	−14.69	6.37	−2.31	0.0211	−57.92	6.68	−8.67	< 0.0001
Hospital	−20.11	6.92	−2.91	0.0036	−39.37	6.86	−5.74	< 0.0001
Clinic, nursing hospital								
Seoul	35.47	5.56	6.38	< 0.0001	25.68	5.53	4.64	< 0.0001
Neighboring city^a^	46.04	4.48	10.27	< 0.0001	32.15	4.46	7.21	< 0.0001
Other cities								
National health insurance								
Medical aid	253.33	25.23	10.04	< 0.0001	232.04	24.84	9.34	< 0.0001
CCI: 0								
CCI: 1	1.70	5.47	0.31	0.7559	−1.99	5.45	−0.36	0.7152
CCI: 2	16.29	6.08	2.68	0.0074	6.63	6.14	1.08	0.2797
CCI: $\ge$ 3	74.42	4.94	15.05	< 0.0001	53.43	5.35	9.98	< 0.0001

^a^Neighboring city includes Busan, Daegu, Incheon, Gwangju, Daejeon, Ulsan, and Gyung-gi.

*CCI* Charlson Comorbidity Index, *Dx* diagnosis-injury of nerves and spinal cord at neck level (S14.x), at thorax level (S24.x), or at abdomen, lower back and pelvis level (S34.x).

### Readmission after the first admission for TSCI and its associated factors from logistic regression analysis

The factors affecting readmission after TSCI are shown in Table 5. Females had a higher readmission than males, and lumbar and sacral spine injuries had higher readmission than other injuries. The 40–59 years age group had the highest readmission and the highest readmission in clinics and nursing hospitals. The readmission was 2.2 times higher for those using medical aid, and the readmission was 1.3 times higher when the CCI was 2 or more.

**Table 5:** Readmission after 1st admission for traumatic spinal cord injury and its associated factor from logistic regression analysis.

Parameter	Univariate logistic analysis				Multivariate logistic analysis			
	OR	95% CI		*P*	Adjusted OR	95% CI		*P*
Male	0.68	0.65	0.72	< 0.0001	0.76	0.72	0.81	< 0.0001
Female	Ref				Ref			
Dx: S14.x	0.58	0.54	0.62	< 0.0001	0.67	0.63	0.72	< 0.0001
Dx: S24.x	0.73	0.66	0.80	< 0.0001	0.79	0.72	0.88	< 0.0001
Dx: S34.x	Ref				Ref			
Age: –19	Ref				Ref			
Age: 20–39	1.16	0.99	1.35	0.0651	1.11	0.95	1.30	0.1847
Age: 40–59	1.33	1.14	1.54	0.0002	1.30	1.12	1.51	0.0006
Age: 60–79	1.19	1.03	1.38	0.0201	1.10	0.94	1.28	0.2374
Age: 80–	0.73	0.62	0.87	0.0004	0.60	0.50	0.71	< 0.0001
Tertiary hospital	0.62	0.57	0.67	< 0.0001	0.84	0.77	0.92	0.0001
General hospital	0.63	0.58	0.68	< 0.0001	0.80	0.73	0.87	< 0.0001
Hospital	0.91	0.84	1.00	0.039	0.97	0.89	1.06	0.511
Clinic, nursing hospital	Ref				Ref			
Seoul	0.91	0.85	0.97	0.0034	0.86	0.81	0.93	< 0.0001
Neighboring city^a^	0.86	0.82	0.91	< 0.0001	0.88	0.83	0.93	< 0.0001
Other cities	Ref				Ref			
National health insurance	Ref				Ref			
Medical aid	2.24	1.55	3.24	< 0.0001	2.18	1.50	3.16	< 0.0001
CCI: 0	Ref				Ref			
CCI: 1	1.22	1.15	1.31	< 0.0001	1.21	1.13	1.29	< 0.0001
CCI: 2	1.38	1.28	1.49	< 0.0001	1.38	1.28	1.49	< 0.0001
CCI: $\ge$ 3	1.32	1.24	1.40	< 0.0001	1.36	1.28	1.46	< 0.0001

^a^Neighboring city includes Busan, Daegu, Incheon, Gwangju, Daejeon, Ulsan, and Gyung-gi.

*CCI* Charlson Comorbidity Index, *Dx* diagnosis-injury of nerves and spinal cord at neck level (S14.x), at thorax level (S24.x), or at abdomen, lower back and pelvis level (S34.x).

## Discussion

TSCI is a devastating event that affects an individual’s quality of life from the moment of injury. Therefore, nationwide studies on the TSCI have been conducted in several countries^5,11^. In South Korea, three large-scale studies on spinal cord injury have been published: one study was on non-TSCI, the other was a study on drug treatment for traumatic spinal cord, and the last study analyzed the burden of work-related TSCI^9,12,13^. However, in previous studies, assessment of surgical treatment in TSCI was not conducted. This study is the first to investigate the trend of surgical treatment for TSCI and analyze the factors affecting readmission and length of stay.

In South Korea, TSCI occurs twice as often in men than women, with an average age of approximately 52 years. Shibahashi et al. analyzed patients with TSCI in Japan from 2004 to 2015^14^. Of the 8,069 patients with TSCI, 78% were male (male:female ratio = 3:1) and the median patient age was 63 years. Dru et al. conducted a study using the US Nationwide Inpatient Sample database from 1998 to 2009 for traumatic cervical spinal cord injury with fracture hospitalizations^15^. Among the total of 44,432 patients, 33,092 (75%) were males and 11,332 (25%) were females. The mean age of the patients was 46 years.

Miyakoshi et al. analyzed the characteristics and incidence of TSCI in Japan in 2018^5^. In 2018, 4603 patients with TSCI were treated. In 2018, the total population of Japan was 126.44 million. In approximately 85% of the cases, the damaged area was located in the cervical region. In the study by Shibahashi et al., the injury locations were cervical (83%), thoracic (9%), and lumbar (8%). Fall on level surfaces was shown to be the most common cause of TSCI^14^. In our study, 30,979 TSCI patients were registered from 2008 to 2020. The diagnostic sites of TSCI were mainly the cervical (69.1%), followed by the abdomen, lower back, and pelvis (22%) and the thorax (8.9%). The areas operated on due to TSCI were cervical in 64.4%, thoracic in 17.2%, lumbar in 18.3%, and sacral in 0.1%. In our study, 23,260 (75%) patients did not undergo surgery, and 7719 (25%) patients underwent surgery; thus, a quarter of patients with TSCI underwent surgery.

In the study by Miyakoshi et al., 1577 of 4423 patients underwent surgery. 35% of patients underwent surgery^5^. Farhadi et al. analyzed patients with TSCI treated at Ohio State University Wexner Medical Center between January 2008 and September 2015^16^. Of the 99 patients, 80 underwent surgery. In this study, we also analyzed the treatment trends from 2008 to 2020 and found that treatment changed as the number of surgeries increased. And trends in surgical methods were also analyzed. Except for corpectomy and fusion, there was a statistical increase in anterior fusion, posterior fusion, and laminectomy surgeries (P < 0.05).

Sreeharsha et al. analyzed the risk factors of morbidity and mortality within 30 days after spinal trauma^17^. The major predictors of mortality after spinal trauma were found to be cervical spine injury, complete neurological disability, chest injury, and ankylosing spondylitis, with older age and thoracic spine injury contributing to higher morbidity and longer hospitalization^17^. Bak et al. analyzed the aspects of cord injury according to the impact of mechanism. It was reported that the possibility of motor sensory recovery from the high-energy mechanism of injury is low^18^. Jian et al. analyzed risk factors for tracheostomy in spinal cord injury. Dislocation, thoracic injury, and ASIA grade A were reported as risk factors^19^. Shin et al. analyzed differences in duration of emergency status. There was no difference in the number of hospitalization days between quadriplegia and paraplegia, and between complete and incomplete injuries^7^. In our study, we analyzed risk factors affecting the length of hospitalization and readmission in patients with TSCI. Risk factors for longer hospitalization were thoracic spine injury, older age, CCI of 3 or higher, and male sex. Risk factors for readmission included age 40–59 years, lumbar and sacral spine injuries, CCI of 2 or higher, and the female sex.

This study has some limitations. First, the HIRA database was built for invoicing purposes, and data may have been omitted or mistakenly entered if a South Korean hospital or clinic did not enter the correct ICD-10 or medical practice codes during examination and treatment.

This may affect the overall outcome regarding the incidence of acute SCI and the development of complications. However, this is a large population-based study, and we believe such errors had little impact on the results. Second, clinical information on pain level, neurological status, quality of life, functional outcomes, radiographic findings, complexity of surgery, and reasons for readmission were not available. Third, the reason for readmission was unknown; thus, it is undetermined whether the readmission was related to TSCI surgery. Finally, this was not a randomized comparative study. Surgical choice may vary depending on the surgeon and facility. However, this study is the first to analyze the treatment method of TSCI using HIRA data and presents useful information to operators and patients by suggesting the length of hospitalization and risk factors for readmission.

## Conclusions

A quarter of all patients with TSCI underwent surgical treatment from 2008 to 2020, and the number of surgeries performed increased annually. The risk factors for length of hospitalization were thoracic spine injury, older age, CCI of 3 or higher, and the male sex. The risk factors for readmission were 40–59 years of age, lumbar and sacral spine injuries, a CCI score of 2, and female sex.

## Data Availability

The datasets generated during and/or analyzed during the current study are available from the corresponding author on reasonable request.
